# Growth of preterm infants at the time of global obesity

**DOI:** 10.1136/archdischild-2018-315006

**Published:** 2018-07-21

**Authors:** José Villar, Francesca Giuliani, Josep Figueras-Aloy, Fernando Barros, Enrico Bertino, Zulfiqar A Bhutta, Stephen H Kennedy

**Affiliations:** 1 Nuffield Department of Women’s & Reproductive Health and Oxford Maternal & Perinatal Health Institute, Green Templeton College, University of Oxford, Oxford, UK; 2 Ospedale Infantile Regina Margherita-Sant’Anna, Città della Salute e della Scienza di Torino, Torino, Italy; 3 Department of Pediatrics, University of Barcelona, Barcelona, Spain; 4 Programa de Pós-Graduação em Saúde e Comportamento, Catholic University of Pelotas, Pelotas, Brazil; 5 Programa de Pós-Graduação em Epidemiologia, Universidade Federal de Pelotas, Pelotas, Brazil; 6 Dipartimento di Scienze Pediatriche e dell’Adolescenza, Cattedra di Neonatologia, Università degli Studi di Torino, Torino, Italy; 7 Center for Global Child Health, Hospital for Sick Children, Toronto, Ontario, Canada

**Keywords:** growth, obesity, neonatology

Preterm birth, 90% of which occurs between 32 and <37 weeks’ gestation,^1 2^ is a complex heterogeneous syndrome interlinked with the stillbirth and intrauterine growth restriction syndromes.^3 4^ Its phenotypes are associated with different gains in neonatal weight,^5^ morbidity and mortality,^6^ and perhaps body composition, growth and development. Preterm birth is related to several aetiologies, although nearly 30% of all preterm births are not associated with any maternal/pregnancy conditions or fetal growth restriction.^6^ This group is, therefore, the target population for constructing postnatal growth standards for preterm infants.^7 8^ There is disagreement, however, about how best to monitor the postnatal growth of such a heterogeneous group of newborns. In fact, a systematic review identified 61 existing longitudinal charts for preterm infants, many with considerable limitations in gestational age estimation, body measurement, length of follow-up and description of feeding practices and morbidities.^9^

The problem requires four fundamental issues to be considered.^10^ 

**First**, size at birth measures (eg, birth weight, length and head circumference), which are taken only once per infant, are a retrospective summary of fetal growth reflecting the intrauterine environment and overall efficiency of placental nutrient transfer. Postnatal growth, on the other hand, requires repeated anthropometric measures after birth, complemented by feeding practices and morbidity data. Therefore, the use of size at birth by gestational age, cross-sectional data taken only at birth to evaluate the postnatal growth of preterm infants cannot be justified either physiologically or clinically. Implicit in the concept of growth is the requirement for repeated measures over time, which can obviously not be captured with a single birth measure. Furthermore, fundamental factors determining the postnatal growth of preterm infants that change over time, such as feeding regimens and organ maturity influencing morbidity, are by definition not included in the data used to construct cross-sectional size at birth charts. Hence, calling such charts ‘postnatal growth references’ should be avoided because the terminology misrepresents the nature of the underlying data used for their construction.

Moreover, using newborn cross-sectional data, in an attempt to reconstruct uncomplicated fetal growth patterns retrospectively, and then pretending that those patterns represent the normative postnatal growth for preterm infants is a massive biological jump that requires accepting the concept that preterm infants should grow like fetuses.^11^ This often-cited concept has not been proven empirically. Fetal growth trajectories are seldom achieved in clinical practice resulting in ‘extra-uterine growth restriction’ being overdiagnosed.^12^ In addition, the metabolic mechanisms are not equivalent as early fetal growth is mostly related to IGF-2 and its effect on placental size and nutrient delivery,^13^ while postnatal growth is modulated primarily by the GH/IGR-1 axis.

Feeding regimens for preterm infants that aim to reach ‘fetal growth’ levels (ie, very rapid weight gain during the first few months of life), and that result in both a transient high proportion of body fat by term-corrected age^14^ and possibly an early childhood adiposity rebound,^15^ could do more harm than good.^16^ Such preterm infants may be accumulating long-term, adverse, health effects, including the appearance in adulthood of components of the metabolic syndrome, and increased cardiovascular risk.^17 18^ Thus, given the limited information available in the literature, it would seem important to implement large, multicentre, randomised trials as a matter of urgency to evaluate feeding strategies for both very preterm and moderate to late preterm infants; the latter group is a rather neglected subpopulation even though it represents close to 80% of all preterms.^19^

**Second**, size at birth *reference charts* are mostly based on routinely collected data, with limited or no standardisation or quality control of anthropometric measures or reliable gestational age estimation, and describe how fetuses *have* grown at a particular place and time (even decades ago). Conversely, preterm postnatal *standard charts*, with prospective measures standardised, gestational age estimated by early ultrasound and childhood follow-up, define how preterm infants *should* grow under optimal conditions considering their degree of maturation.^8 20^ The terms ‘reference’ and ‘standard’ should not be used interchangeably because they are based on different data and have different objectives.

**Third**, ‘distance growth’,^21^ which represents the value attained at specific postnatal ages, is the most robust and commonly used tool in clinical practice. Velocity growth expresses the change in value of an anthropometric measure taken on the same infant between two time periods,^22^ that is, it requires individual,^23 24^ repeated data.^8 20^ Hence, the need for more than one measure across time on the same individual is germane to the growth velocity concept.^22^ Cross-sectional size at birth data, by definition, cannot estimate velocity growth.

In some clinical settings for practical reasons, velocity fixed rates of 15 g/kg/day, 10–30 g/day for weight or 1 cm/week for length are used. This is problematic because the 15 g/kg/day constant weight gain value is neither biologically plausible nor supported by longitudinal data. An alternative is to evaluate weight gain as g/kg/day^25^ at different postnatal ages. However, this format provides a misleading picture of newborn growth kinetics by describing an earlier peak of weight gain that has limited contribution to the total weight increase.^5 26^ The weight velocity of these tiny babies, if it is going to be used, is better described as weight changes in g/day according to postnatal age, which follows a non-linear pattern.^5 26^ Considering the methodological issues, difficulties with calculation, interpretation at the bedside and that most units routinely just plot anthropometric measures according to age, distance charts are preferable to velocity growth measures.

**Lastly**, postnatal charts for preterm infants should include weight, length and head circumference taken from the same newborn population. This is a problem for size at birth charts derived from combining studies.^11^ For example, only two^27 28^ of the six data sets included in an often employed meta-analysis^29^ had all three measures taken from the same infant. Therefore, when these charts are used to evaluate a newborn, its weight is compared with one population but its length and head circumference (very relevant for developmental risk) are compared with a different population.

Considering these conceptual issues, the INTERGROWTH-21^st^ Project has produced international standards for the postnatal growth of preterm infants that complement the existing WHO Child Growth Standards, which are only for term newborns. We enrolled, for the first time, ‘healthy’ pregnant women initiating care <14 weeks’ gestation to study, in the same sample, fetal growth,^30^ newborn size,^31^ and body composition,^32^ the postnatal growth of preterm newborns,^8^ and the follow-up of all these babies up to 2 years of age,^33^ including neurodevelopmental assessment.^34^ The project used the same equipment, standardised methodology and feeding practices based on human milk, to produce the first integrated set of international standards for monitoring fetal and newborn growth and development. Detailed descriptions of breast feeding patterns and the introduction of complementary feeding are presented elsewhere.^35^ The project matches the WHO Multicentre Growth Reference Study because it adopted the same prescriptive approach in selecting pregnant women at population and individual levels,^36^ and because the newborn anthropometry overlaps with the WHO Child Growth Standards perfectly.^37 38^ Exactly the same prescriptive approach was applied to construct^7^ the longitudinal INTERGROWTH-21^st^ Preterm Postnatal Growth Standards.^8^

Finding that healthy pregnant women receiving adequate healthcare can achieve a preterm birth rate as low as 4.9% has established an evidence-based target for perinatal programmes. However, it meant that fewer preterms were born than originally estimated, mostly because of the reduction of very preterm births.^8 10^ Hence, the international INTERGROWTH-21^st^ Preterm Postnatal Growth Standards are robust standards from >32 to 64 weeks’ postmenstrual age for >90% of the preterm babies born worldwide. They provide, even at low gestational ages, consistent smooth centiles that follow a logical pattern,^8^ based on repeated measures directly relevant to how preterm *infants* should grow. Supporting e-learning courses for the global standardisation of growth monitoring of preterm infants are freely available at: https://globalhealthtrainingcentre.tghn.org/intergrowth-21st-course-maternal-fetal-and-newborn-growth-monitoring/ https://globalhealthtrainingcentre.tghn.org/preterm-infant-feeding-and-growth-monitoring-implementation-intergrowth-21st-protocol/.

**Figure 1:**
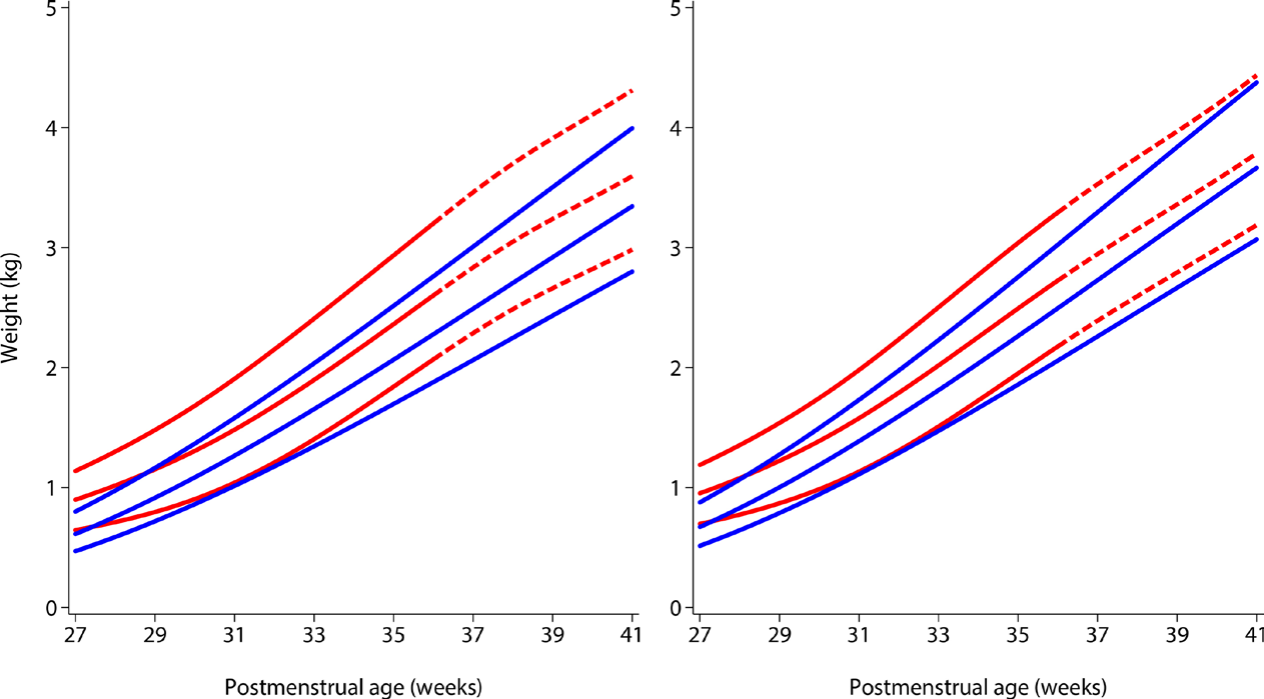
Comparison of 10th, 50th and 90th centiles of the INTERGROWTH-21^st^ Preterm Postnatal Growth Standards for weight^8^ (solid blue lines) with the Fenton size at birth charts^26^ (solid and dashed red lines) according to sex (left, girls; right, boys) from 27+0 to 40+6 postmenstrual weeks. The dashed red lines correspond to the gestational ages at which the Fenton charts were modelled to reach the WHO Child Growth Standards at 50 weeks (figure modified from Villar *et al* 10).

Which type of chart is selected for growth monitoring has a profound effect on the clinical care of *all* preterm infants. Figure 1 compares the international INTERGROWTH-21^st^ Preterm Postnatal Growth Standards^8^ for weight with the Fenton size at birth charts (the latter developed using cross-sectional birth data from studies of newborns without postnatal follow-up) at the 10th, 50th and 90th centiles from 27 to 40 weeks^29^; a longer and detailed comparison is discussed elsewhere.^10^

Despite similar slopes, size at birth charts are consistently higher than the INTERGROWTH-21^st^ Preterm Standards up to term, that is, preterm infants’ growth is not that of fetuses, which is reflected by Fenton charts. Very reassuringly, the INTERGROWTH-21^st^ centiles have smooth patterns consistent with the remainder of the follow-up period. This is because the centiles in the INTERGROWTH-21^st^ standards are based on 1759 repeated measures (equivalent to a cross-sectional study with double the sample size) taken across the follow-up period up to 64 weeks,^8^ not just the values at 27 weeks.

These differences have major clinical implications: (1) size at birth charts diagnose more ‘extra-uterine growth restricted preterms’, many of whom are healthy and growing adequately along their centile, yet they now require ‘treatments’ and nutritional support, and (2) if it is expected that preterm infants should reach fetal weight levels, as required by size at birth charts, they must be fed more than the recommended human milk-based strategy because presently they seldom reach such weights. This strategy forces preterm infants to weight and body composition levels disproportionate to their length, that is, they become overweight for length at discharge, which increases the risk for chronic diseases,^17 18^ but may not improve developmental outcomes.^39^ Is this the goal for preterm infants at the time of the global obesity epidemic? We do not believe so: the global prevention of obesity, cardiometabolic syndrome and related complications in preterm infants should start at the incubator.
